# Social isolation and exclusion: the parents' experience of caring for children with rare neurodevelopmental disorders

**DOI:** 10.1080/17482631.2020.1725362

**Published:** 2020-02-12

**Authors:** Genevieve Currie, Joanna Szabo

**Affiliations:** School of Nursing and Midwifery, Faculty of Health, Community and Education, Mount Royal University, Calgary, Alberta, Canada

**Keywords:** Children, rare disease, neurodevelopmental disorders, chronic disease, parents, caregivers, healthcare, experience of healthcare

## Abstract

**Purpose**: The experiences of parents caring for the complex care needs of children with rare neurodevelopmental disorders are not well understood. Parents struggle to meet their children’s medical, behavioural, and social needs within and across health, social, and family systems. The purpose of this study was to explore the parents’ experience of caring for medical and social care needs for children with rare neurodevelopmental disorders.

**Methods**: Hermeneutic phenomenology was used for the data analysis. Fifteen parents participated in semi-structured interviews.

**Results**: Interpretive analysis revealed four insights: (a) difference in children’s behaviours and disease manifestations led to misunderstanding and vulnerability within social domains, (b) social taboo and stigma were experienced with rarity, (c) fragmented disconnected care from health and social systems impacted families, and (d) incomprehension from friends and family occurs when managing daily care.

**Conclusion**: New interpretations and increased understanding of parents’ experiences are required in supporting parents caring for children with complex needs. Understanding parents’ experiences could reduce social isolation and exclusion, and mitigate appropriate and supportive practices and services within and across medical, social, and family systems.

## Introduction

Parents’ experiences of caring for the medical, behavioural, and social needs for children with rare neurodevelopmental disorders (NDDs) are largely misunderstood and unknown. In particular, the parents’ experience of managing complexities of care within health, social, and family systems remains under-researched. Children often experience complex physical and behavioural challenges, compounded by rarity of disorders and lack of knowledge about the disease. They require multisystem involvement and integrated care for managing the disorders in the community. These challenges contribute to intensive parenting responsibilities. Narratives of parents largely remain unvoiced because of fear of social misinterpretation, shame, and misunderstanding (Carpenter & Austin, 2007). These realities contribute to the untold stories of parents who care for children with complex disorders. Within this study, parents were asked to share their experience of caring for children with rare NDDs surrounding medical and social supports. Parents disclosed their experience of isolation and exclusion within medical, social, and family systems.

Rare diseases affect approximately one out of 12 Canadians, with two-thirds being children. A rare disease is defined in Canada as a disease that occurs in one in 2,000 people (Rare Disease Foundation of Canada, n.d.). Rare NDDs are often caused by a single gene mutation or genetic variants in several genes (Niemi et al., 2018). Rare NDDs encompass diseases of the brain and neurological system leading to seizures, physical and developmental disabilities, learning and cognitive disabilities, mental health, and psychiatric disorders. Behavioural challenges can include difficulty with emotions, communication, social norms, anger, aggression, repetitive behaviours, and self-injurious behaviours resulting in lack of independence, and intensive caregiving (Einfeld, Ellis, & Emerson, 2011). Children with rare NDDs have more severe disabilities than children with more common NDDs, such as autism and attention deficit hyperactivity disorder (Bishop, 2010). Overall, children with NDDs utilize health care services more significantly with three times greater the number of hospitalizations and two times the number of physician visits than the general paediatric population (Arim et al., 2017). Children require frequent supports from inpatient and outpatient medical, and community-based systems, including primary care, emergency care, rehabilitation, and government disability support (Arim et al., 2017; Diallo, et al., 2018; Miller, Shen, & Mâsse, 2016). However, current practice models provide disjointed fragmented care because of highly compartmentalized health and social systems (Brodeur, Chouinard, & Hudon, 2017).

There are only a limited number of studies on the collective experience of parents caring for children with rare NDDs navigating within systems. The literature discusses rare diseases, rare NDDs, and the experience of parents with children with NDDs or specific rare diseases. Highlights from the literature will be discussed next.

Parents caring for children with NDDs experience more medical, psychological, and familial dysfunction because of the additive nature of children’s NDDs and disease manifestations, leading to long-term exposure to stress and caring responsibilities (Lach et al., 2009; Miodrag & Hodapp, 2010). Parents experience higher levels of stress with NDDs, particularly from children’s emotional and behavioural challenges than with other developmental disabilities (Craig et al., 2016). Mothers of children with NDDs experience declining health at a faster rate than fathers (Burton, Lethbridge, & Phipps, 2008). These medical and psychological challenges perpetuate more discrimination and isolation for mothers when they are judged as not coping with the demands of being caregivers (Carpenter & Austin, 2007; Robertson, 2014). As a result, mothers may not share their struggles and difficulties with providers and family for fear of misunderstanding and judgement (Carpenter & Austin, 2007).

Parent experiences in caring for children with NDDs and challenging emotional behaviours are not always situated in conventional parenting practices (Williams & Murray, 2015). Mothers provide most caregiving for children with developmental and intellectual disabilities, including collaboration of care needs with physicians and community-based care (Lafferty, O’Sullivan, O’Mahoney, Taggart, & van Bavel, 2016) and thus are more susceptible to stress and burnout. Williams and Murray (2015) reported mothers become oppressed and marginalized when caring for children outside the traditional discourses of motherhood. Mothers and their children learn to navigate in the world as the “other,” with this exclusion being true for both mothers and their children. Parents caring for children with disabilities experience intrapersonal, interpersonal, and structural constraints that are amplified and sometimes require substantial effort to navigate (Williams & Murray, 2015). These unrealistic parenting expectations can lead to disconnection and vulnerability (Brock, 2014; Fernandes & Robertson, 2019; Robertson, 2014).

Parenting expectations include parenting a “good” child. Carpenter and Austin (2007) described the experiences of mothers caring for children with complex NDDs such as attention deficit hyperactivity disorder. These authors shared that children with behavioural differences disrupt the popular “text” (Carpenter & Austin, 2007, p. 660) of mothering, and these atypical experiences of motherhood are pushed to the margins. Mothers try to engage within social arenas and social practices, but they are constrained by cultural norms and standards of the good child and good mother (Robertson, 2014). If the child is exhibiting aberrant behaviour that is socially sanctioned, then families could experience shame and marginalization (Carpenter & Austin, 2007).

Mothers of children with NDD also experience a sense of disregard and disengagement by medical professionals, friends, and immediate family (Austin & Carpenter, 2008). Mothers described constraints when accessing care, support, and assistance with their children’s atypical and unknown manifestations of disease, requiring multisystem involvement. When parents raised issues to healthcare providers (HcPs), they felt shunned, dismissed, and uncomfortable because of medical inexperience and lack of knowledge in handling complex medical and behavioural issues (Fernandes & Robertson, 2019). This led to poor resolution of medical issues and few supports for families. Children also experienced rejection because of troublesome, disruptive behaviour outside of social expectations or codes of conduct. Austin and Carpenter (2008) described the maternal narratives as socially “troublesome” (p. 390) and mothers being considered “troubled” (p. 390) because they were not managing the issues with their children effectively.

As evidenced by the literature reviewed, there is a need to uncover and reveal narratives of the lived experience of parents caring for children with complex medical and behavioural manifestations with rare NDDs. New understandings and interpretations of the experience of caring for children who require multisystem involvement can offer insights into parenting experiences to mitigate appropriate supports and services for families. Therefore, the purpose of this study was to explore the parents’ experience of caring for medical and social care needs for children with rare NDDs.

## Method

Family systems theory (Friedermann, 1995) and general systems theory (Von Bertalanffy, 1968) informed the research question and interview questions regarding parent experiences of health and social support. Within these theories, families are viewed as units which interact, adapt, participate and influence providers, systems, and structures (Friedermann, 1995).

Our research question asked: What are parents’ perceptions and experiences of support from health and social services communities when living with a child with a rare NDD?

### Methodology

Following the philosophy of Gadamer (2013), we utilized hermeneutic phenomenology to understand what has been misunderstood and/or requires new interpretations surrounding the parent experience of caring for a child with a rare neurodevelopmental disorder (NDD). The process of interpretation, revealing, and understanding other’s experiences liberates prejudgments and assumptions (Gadamer, 2013). Gallagher (1992), a hermeneutic phenomenology researcher, suggested that researchers’ preunderstandings or assumptions are “continually being modified by experience; they can be radically altered and corrected as they proceed to understanding” (p. 69). Therefore, hermeneutic phenomenology can enrich and broaden people’s understanding of the reality of parenting a child with a rare NDD. Both researchers worked with children and parents with NDDs as paediatric nurses. One researcher is a mother caring for a child with a rare NDD. Both researchers had considerable experience with exploring phenomena which are not well understood. While conducting a hermeneutic phenomenology study both researchers became immersed in the parents’ experience and were open to understanding new experiences and understandings. Openness to lived experience incorporates an expanded understanding of parents’ social worlds, culture, history, and challenges, so there can be a fusion of new understandings and horizons (Gadamer, 2013). Gadamer (2013) suggested, by sharing suppressed or silenced experiences, an intermingling of narratives beyond the present discourse can occur while reaching for common ground.

### Recruitment and participants

Through hermeneutic phenomenology, we aimed to understand the meaning of caring for children with rare NDDs by engaging parent participants. Following Creswell’s (2013) recommendation, we used purposive sampling to recruit 15 parents (11 mothers and four fathers) from medical genetic, endocrine, and neuropsychiatry clinics and received self-referrals from parent support groups within several Western Canadian hospitals. Smaller sample sizes reach adequacy when expanding understanding through interpretation (Smith & Osborn, 2008), which is typical for qualitative inquiry that does not aim to generalize findings (Sandelowski, 1995).

Eligibility criteria required participants’ children to meet the definition of a rare NDD (defined as having a prevalence of below 1 in 2000 live births) set out by the Rare Disease Foundation of Canada (n.d.). Parents were between 30 and 45 years of age with children 11 years of age and younger, who were diagnosed with a range of rare NDDs within 2 years of birth. One family had two children with the same rare NDD. All children had two or more comorbidities because of NDDs. Parents had high school education with the majority having postsecondary education. Fourteen of the parents were living with a partner; one was a single parent because of divorce. Four of the parents were married to each other and were interviewed separately to provide individual perspectives. Parents had middle to higher income status, with more than one-third of the mothers and all fathers employed. Five of the parents were living in rural settings.

### Data collection

Prior to commencing the semi-structured interviews, verbal and written informed consent was obtained (as part of the project’s certification with the university human research ethics board). Anonymity was provided for the participants as well as access to supportive counselling from an outside agency. Participants selected the setting for the semi-structured interviews, and interviews were conducted in private settings within two urban centres and several rural areas in Western Canada. We conducted in-depth, face-to-face interviews that were 45–120 min in length using open-ended and flexible questions with each of the participants. Topics included parents’ experiences with health, social, and family supports, initial diagnosis of the rare NDD, and ongoing care within hospital- and community-based supports.

### Analysis and rigour

As researchers, we transcribed verbatim the audio-recordings of the interviews and, following the methodological considerations of Moules a hermeneutic Gadamerian researcher (2002), we also engaged in analysis both individually and collectively, by reading, reflecting, and interpreting in multiple iterations the transcripts and notes. We dwelt with impressions, alternative explanations, diverse patterns, and insights through both narrative and interpretive lenses (Moules, 2002; Whitehead, 2004).

### Ethical considerations

The study was approved by the Human Review Ethics Board (HREB approval #2016-35). The research conformed to the ethical principles for medical research on human beings as set out by the Tri-Council Policy Statement (CIHR, 2018). The study fulfilled requirements for research (information consent, anonymity, and safety of the participants). The study was guided by ethical principles of autonomy, beneficence, non-maleficence, and justice. Participation was confidential and data collection insured anonymity for the participants. Participants could withdraw from the study at any time.

## Results

New and diverse layers emerged in the parent narratives regarding involvement with social, medical, and family systems because of complex disease and behavioural issues. Parents experienced difficulties managing care of their children within and across systems. Four insights emerged from interpreting the parent narratives. These insights were themed (a) difference and vulnerability, (b) social taboo and stigma, (c) systemic dis-ease, and (d) incomprehension. Additional findings considering the parent experience are found elsewhere (Currie & Szabo, 2019; Currie & Szabo, 2018). Throughout this section, the participant codes Parent 1 through to Parent 15 are used to ensure participant anonymity.

### Difference and vulnerability: “they’re not different; they are our kids”

Parents spoke of difference and lack of understanding within common social settings such as playgrounds, shopping centres, and schools when their children exhibited difference from other children. Sometimes lack of understanding came from others in the form of questions or curiosity about difference: “When he was little I got overwhelmed with having to explain everything over and over again” (Parent 1). Another parent shared the experience of her child being different with a lifelong condition: “Everyone around us would say, give him a year or two and all kids catch up. I wanted them to just stop saying this was going to be fine. I know medically this isn’t going away” (Parent 5). In some instances, participants expressed they were uninvited and unwelcomed in some public domains where difference was not tolerated: “They [our children] look typical, they can walk on their own; a lot of the time it’s the looks of, why are you acting like that? … A lot of families don’t understand (Parent 2).

Parents spoke of how their experiences of parenthood were unique from mainstream parenting practices, resulting in social difference.

Your son isn’t reaching milestones, and this changes your perception on parenthood. People think it’s difficult but don’t know what difficult actually is. You can’t blame them. If they don’t have to worry about it, why would they? … I have to live with it and figure it out. (Parent 13)

These narratives can be understandable since the general public has little exposure to children with complex physical symptoms. cognitive delays and behavioural issues. Unfortunately, this lack of public awareness and exposure to symptoms and behaviors associated with children with rare NDDs reinforced to parents their children were different. When a father was asked about his experience with interacting in public spaces with his son with complex needs, he replied, “I find it awkward. I don’t think they judge you. … They don’t know how to talk to you. Ask me questions. I’d rather you ask questions than stare at him” (Parent 13). One mother relayed her strategy for managing people staring at her child: “I have a t-shirt that I wear for fun and it says, Stop Staring!” (Parent 10).

These differences in children with rare NDDs also led to vulnerability in everyday life from physical threat with disease manifestations and comorbidities. “It’s the juxtaposition of our lives bifurcating instant by instant and people not getting both the normality and abnormality of life” (Parent 14). A mother relayed her incapacity to cope with her child’s medical condition when her husband was away. This narrative spoke to the vulnerability of everyday situations and what might tip the balance: “He’ll be gone for a few days and this is not something I can do by myself. I cannot manage by myself. It there’s an emergency, I definitely cannot manage by myself. I am terrified of him going” (Parent 15). Another mother voiced the constant crises experienced from difference and vulnerability:

We are at the park having a great time and boom, she has a seizure. If we don’t stop it in time, then we are in the hospital. She’s potentially losing brain cells and oxygen. (Parent 15)

Parents relayed they felt they were in a movie, watching something that could not possibly be their lives. However, they were centre stage, managing complications, and disease manifestations they had never imagined. Some parents expressed uncertainty from anticipating ill-fated outcomes from the rare disease with no reprieve. “We were given the diagnosis of the short life expectancy and that he doesn’t breathe well at night, that makes us live in anticipatory grief that people don’t really get” (Parent 8). Another parent said: “For me having a child that has neurological disorder has been a scary thing because it involves the brain. … There’s always this uneasiness that comes along with knowing that your child’s brain is not typical and because the other things are unknown” (Parent 5). A third parent described the possibility of the disease getting worse: “Dealing with the unknown is the worst, wondering if and when things are going to come [disease symptoms] and what they are going to be” (Parent 7).

Some parents expressed they never knew what would happen next. Parent 4 stated, “It’s not like they [medical incidents] are one-time events. They are a repetition. You’re exhausted because you are always running.” When a father was asked if there was something he would like to say to other parents of children with rare NDDs, he mentioned a sense of difference:

Don’t look at other people’s kids. When he was eleven months old, I took him to daycare, that’s when it hit home. He’s really behind. I came home and cried for two days. Focus on your own path. … Don’t compare. (Parent 13)

Thus, parents spoke of the experience of social and medical differences as well as uncertainty and vulnerability with their children because of disease manifestations and complexities associated with rare NDDs.

### Social taboo and stigma: “it’s rare; it’s not taboo.”

Parents experienced social taboo and stigma surrounding rare diseases when interacting with families who had neurotypical children. Stigma occurred when invisible disabilities confronted the artifice of societal norms. One parent stated, “We need awareness and people to understand. It’s rare; it’s not taboo” (Parent 1). Another parent shared the following story:

This is the only time I’ve ever lost it in public. A mom at IKEA told me to get my kid under control. He was having a seizure, and lots of kids get it [seizures with this medical condition].

She said, “Get your kid under control. He’s disturbing our lunch.” She said, “It’s not fair to others to bring your kid to a public space. It’s not fair that my kid can’t eat his food.”

I could feel myself getting hotter. I yelled back, “Screw you! He’s having a temper tantrum because he wants to. His brain exploded in his head and he can’t help it.” (Parent 1)

These incomprehensible and unfathomable social situations revealed stigma from others about managing medical complexity and disability within public arenas. Parents spoke about standing up for their children because of social discomfort, but at great emotional cost: “You’re a mother bear anyways, but when your child has any kind of struggle, you have this hair-trigger reaction if someone just looks at your kid the wrong way. It can destroy you” (Parent 4).

Parents expressed guilt because of difficulties and challenges of caring for their children and reaffirming love for their children. Revealing these struggles often led to misunderstanding and social stigma. There was a sense of taboo when parents discussed unconventional parenting struggles. A mother described having to hold down her child to get bloodwork:

Sometimes I come across as harsh. We need to get this blood test, and we can sit around and talk about it or we can just get it done. It sucks for her and she doesn’t understand. It’s just part of taking care of her. … I just know what we have to do. (Parent 12)

And another parent discussed discomfort in revealing the real experience:

It’s difficult just talking about it with other people; it’s awkward. They don’t understand. They feel I’m holding something back, like I don’t talk about my kids as much as they talk about theirs. In general, we just keep to ourselves more. (Parent 3)

Parents also described disconnection and a sense of social taboo from other parents who had “typical” developing children.

It can be very stressful to have him in circumstances where I’m not comfortable with the parents and the kids who are there and who don’t know our story. I try to avoid all circumstances with kids that we don’t know well, and I’m worried things will happen, and I’ll stand there like the embarrassed parent trying to explain why this is happening. It’s very isolating. (Parent 5)

There was also a sense of lack of reciprocity between parents of a child with a disability and those with a neurotypical child. As one mother described,

My complaint about people is when they complain about things that I wish we had. Like having to drive their kid to soccer or dance recital. … . They’re not trying to be rude, they are trying to make conversation, but it’s really hard. You wish you could have those experiences. (Parent 8)

The same mother extended this thought further: “Other people have other things to talk about like their child’s ballet recital and we can’t relate to each other” (Parent 8). Parent 1 discussed the social discomfort when interacting with other families:

There’s a kid that goes to the same preschool, and I went up to a mom and said, “You know what it’s like to walk behind your kid carrying a backpack. Mine is a feeding tube and yours is oxygen. We walk through the line of parents and no one says anything to you, but they all watch you walk your kid. They are all looking. … You have to get over your self-consciousness.” (Parent 1).

One parent described social discomfort when her son displayed aberrant behaviour:

It is difficult if my daughter wants to go to the park and he [the child with an NDD] is there with us. There will be problems if he wants to be included. He acts out. It’s hard to have kids into the house. (Parent 5)

In summary, parents expressed breaking social taboos in public arenas when their children displayed atypical behaviours. This led to avoidance of social situations and stress for families.

### Systemic dis-ease: “I felt helpless … ”

All parents expressed frustration because of systemic barriers with episodic care models and lack of coordination services within medical and government systems. “Every time you meet with a clinic or hospital, you’re starting at Day 1 again” (Parent 3). A parent shared a fragmented unsafe response from a care provider when she reached out for support: “I spoke to the genetic counsellor on the phone and called her when my son talked about killing himself. She didn’t refer me anywhere” (Parent 11).

Parents described the current system of healthcare with children accessing subspeciality experts with little focus on overall multisystem disease manifestations from rare NDDs. There was generally a lack of discussion or coordination across systems or sectors when determining the plan of care for the child.

My experience is that if my son presents with pneumonia, they’ll look at the pneumonia, but they don’t look at why he has pneumonia, what else within his syndrome is causing it. They don’t put the puzzle pieces together. They look at the pneumonia in isolation of everything else. They have to look at the whole child, all the pieces of the child. (Parent 2)

This uncoordinated care approach did not accommodate children and families with complex care needs from rare disorders requiring long-term intensive supports. A parent provided a vivid narrative of a visit to an Emergency Room, highlighting fragmented management of care across medical and community-based systems:

I was just at the hospital recently because he sprained his ankle. I took him to the ER [Emergency Room]. … I tried to tell him [the physician] my story. I have a kid who can’t walk, and he goes to a middle school with stairs, he has cerebral palsy and gross motor challenges. What do I do? How do I manage this? His answer was, “Well, kids heal pretty fast. You could probably go to Wal-Mart and get one of those lace-up braces.” *…* I couldn’t even send him to school because he couldn’t walk properly. … I went to Wal-Mart, and they don’t make a brace small enough for him. I can’t tape him because of his sensory issues. There I am in an Emergency Room at [the] Children’s Hospital where they have his complete history, and they aren’t willing to help us. … . I felt helpless. I knew my child was hurting. (Parent 5)

Children required multiple supports from providers within medical and social support systems. One mother relayed how these experiences highlight providers' misunderstanding of the range of manifestations with rare NDDs. “Trying to get people[providers] to understand what he has is really difficult. Nobody knows about it. It’s a spectrum” (Parent 1). Another mother highlighted disconnection between the child’s emergent medical symptoms and the supports required within the community:

Do you think a cast might be a better solution? “No, this is how we manage it.” I take the splint off and he falls down the stairs. Now his arm is hanging there. I called my husband. He is going to need to have this reduced. It’s broken bad. He will need an anesthetic, and I need you to come. I can remember driving to the hospital …, all I could think was, “They think I am abusing my child. This is his second broken arm in two weeks. They will think I beat him.” Somebody should have listened to me and put him in a cast in the first place. I have a child that has mobility issues. (Parent 5)

A parent summarized a sense of being diminished and disregarded by the medical system and those providing care.

There have been the few, like the nurse and neuro resident, that have taken an interest in us. In general, [I am] just one more person going through and making demands of them. That’s what it feels like anyways. (Parent 8)

Parents also experienced systemic fragmentation from government disability services. One participant stated, “You need to ask for the right things; they aren’t going to suggest things for you” (Parent 2). Similarly, another parent said, “You have to figure it out on your own; what you can access and the support you need, you get on your own” (Parent 3). A third parent described a lack of transparency regarding available supports and services within the community:

If you don’t know what’s available, you don’t know what to ask for. It’s not enabling me; it’s empowering me. Tell me what the options are and ask me if they would be helpful to me or not. Telling me that listing the options is enabling me is not helpful. I get if you tell people what’s available, you will have to provide a lot more support than you think you need to or it will be a drain on the system, I get that. (Parent 15).

In summary, parents experienced mismanagement and fragmented care within and across health and social care systems. Fragmented care delivery increased the burden of care for families.

### Incomprehension: “very few people get what our life is like”

Parents expressed misunderstanding and lack of comprehension from family and friends about the reality of caring for their children. Instead of overcoming adversities and displaying resiliency as within normative narratives, parents revealed the struggle with coping with day-to-day experiences. “It’s very isolating. People don’t get how exhausting living in high alert is. At any second, things can change and there’s no way to convey to other people what that’s like” (Parent 15). One parent described the daily struggles that are anything but typical:

It is so complicated, and little is known that you are always scrambling and desperate to do the best for your kid, but you don’t know what path to go down. … You feel very overwhelmed and helpless. You are always grieving. The guilt that comes from grieving can be crippling. (Parent 4).

One mother summed up her desire for openness from others about her family’s unique experience with having a rare NDD and wishing for a community of support:

I wish that the world could know how daunting it is [the experience of disease]. You come across some who get it. To be in this place where you are completely overwhelmed; this is not what you wanted for your precious child. I wish there was more of a community. (Parent 5)

Another mother described that friends and co-workers want to be supportive, but they don’t understand. “I have some awesome friends who are helpful. I don’t think they understand. … You’ll have someone come to work saying their dogs were up last night because they ate a bad bone and you’re like, really? You have to laugh or you’ll cry. I haven’t slept in how many years so my compassion for you is zero.” (Parent 12)

Parents described their caring experiences as discredited because of little understanding from family members. “There’s a lack of awareness and lack of understanding even from people who are close to us” (Parent 8). Participants expressed that family members did not comprehend the vigilance and pervasive efforts required in managing the rare NDD disease processes and behavioural outbursts. “Managing her medical stuff is like a full-time job. Appointments, booking appointments, managing medications. I have a full-time job, so balancing is a bit crazy. People don’t realize that it’s all consuming” (Parent 15). A father echoed this experience: “Very few people get what our life is like” (Parent 14). A mother described lack of understanding from family members regarding day-to-day challenges:

People don’t get it; even our family members don’t get it. They don’t know what to call what he has. It’s hard for people to wrap their minds around that. That’s a big problem, and people like easy things that fit into a box. That has been a day-to-day challenge; not having something you can relate to [with family members]. (Parent 8)

Thus, parents experienced incomprehension from family and friends regarding intensive care responsibilities and accountabilities. This incomprehension led to a general sense of marginalization.

## Discussion

This is one of few studies revealing the overwhelming experience for parents managing complex care needs for children with rare NDDs within medical, social and family systems. The narratives illuminate something different and distinctive about parenting a child with a rare disorder with multiple comorbidities. The findings align with family system and general system theory, which acknowledges the impact on families as they intersect and interact with providers, structures and systems (Friedermann, 1995; Von Bertalanffy, 1968). Parents experienced social isolation and exclusion when they bumped up against challenges and constraints within social, medical, and family systems.

Within this study, parents described social exclusion and marginalization within public domains when their children displayed atypical behaviours with outbursts and disruption of a predetermined invisible social norm. This is congruent with Brock (2017) and Wagner, Wilson, Smith, Malek, and Selassie (2015) who described constraints for parents when trying to navigate public arenas because of social stigma and taboo from behavioural manifestations of disease. Parents described not meeting the expectations of parenting “good” children (Austin & Carpenter, 2008; Carpenter & Austin, 2007). Social storylines within popular culture depict parents rising above challenges and overcoming complex care responsibilities with their children. These constructs can release systems (health, social, and family) from understanding care needs and supports required by families and places the onus of responsibility on the parents (Knight, 2013). Families must not only manage difficult home and community situations but be resilient and redemptive while caring for a child with medical and behavioural challenges (Knight, 2013; Muir & Strnadová, 2014). Parents can become further marginalized and excluded if they are not seen to possess resilient traits and characteristics (Fernandes & Robertson, 2019).

Parents, surrounded by a culture of “taking care of your own” (Caldwell, 2007, p. 558), relayed incomprehension and isolation from neurotypical families as well as their own extended families. Brock (2017) and Caldwell (2007) addressed the dominant narratives within families that parents must meet their children’s care needs as part of accountability and responsibility for having children with special needs. In this way, parents too become disabled, overwrought, and destabilized trying to meet their children’s complex needs, facing the discriminatory practices of social isolation that their children are encountering (Ryan & Runswick Cole, 2008).

Parents in the study also expressed vulnerability and exclusion in and between a liminal space in which they could not access normalcy or acceptance of uniqueness (labelled abnormality). Some parents were not comfortable disclosing feelings of being overwhelmed or struggling meeting pervasive care challenges. This has rarely been described in the literature (Lopes, Koch, Sarrubbi-Junior, Gallo, & Carneiro-Sampaio, 2018). Furthermore, challenges with accessing community and family supports due to a paucity of services led to further exclusion for families (Barnert et al., 2017; Brock, 2015).

Parents also struggled navigating within dominant hierarchical systems and power imbalances with healthcare and government disability support providers. In particular, parents in this study described being excluded because of insufficient care modalities not addressing medical and behavioural manifestations associated with the rare NDD. This is congruent with finite studies on parents’ experiences with rare diseases (Galpin et al., 2017; Pelentsov, Laws, & Esterman, 2015). Compartmentalized care models within health and government supports are designed for periodic health incidents, not for complex disease trajectories such as complex rare NDDs (Grut & Kvam, 2013; von der Lippe, Diesen, and Feragen (2017). With unmet care needs, parents experienced pervasive demands and atypical family functioning. Additional resources and supports such as care coordinators are required to navigate these challenging systems and reduce isolation and exclusion (Barnert et al., 2017; Baumbusch, Mayer, & Sloan-Yip, 2018; Thomson et al., 2016). Care coordinators within the care coordination project at the Alberta Children’s Hospital and Boston Children’s Hospital (Antonelli, Browning, Hackett-Hunter, McAllister, & Risko, 2012), support parents with difficult care transitions and link systems and services to manage difficult care needs. Families receiving appropriate social, psychosocial, and financial supports can more easily navigate challenges and tackle the uniqueness of their family barriers (Craig et al., 2016; McConnell, Savage, & Breitkreuz, 2014; Pordes, Gordon, Sanders, & Cohen 2018). Parents become more resourceful and constraints are reduced with medical and social supports from care coordinators and multidisciplinary providers and specific care models and pathways for rare NDDs (Craig et al., 2016; Europlan, n.d.; McConnell et al., 2014; Pordes et al., 2018).

The findings of this study expand new beliefs and perspectives of what is familiar and unfamiliar within the larger understanding of parenting a child with complex medical and behavioural needs. There is a necessity for providers to collectively expose and validate stories of families’ day-to-day struggles and promote new narratives and reinterpretations of parenting for parents of children with chronic disease and disabilities (Harter, Pangborn, Ivancic, & Quinlan, 2017; O’Reilly, 2008; Oliver & Barnes, 2012). This is a move away from what Frank (2013) called the restitution narratives in which hope is tethered to a cure and restitution and resilience are tied to a return to a previous state or normalized expectations. In contrast, parents expressed illness narratives where there is no cure, or change in prognosis with their children’s illness. Frank’s quest narratives are more in alignment with our findings where participants express their struggles when experiencing a difficult illness and reflect on what can be learned from difficult situations (2013). Within systems, providers can support families by addressing the unique needs and values of families; providing supports with ongoing behavioural and emotional challenges; and, assisting families with stress when there is no release from hypervigilant caregiving (Craig et al., 2016; Fernandes & Robertson, 2019; Snowdon, Schnarr, & Alessi, 2014).

Parents spoke of detachment, fear, and stigma from others, as their children were perceived to be socially and medically different because of manifestations from a rare NDD. A focus on the rarity of the disorder as abnormal, atypical, and unknown can dismiss the experiences and problems of families as challenges that cannot be resolved or improved. This can lead to further isolation for families socially and personally. Collaborative care models with families are necessary to develop appropriate health and social care policies and care practices for rare disorders (Rabeharisoa et al. 2014). We suggest opening the dialogue with parents to understand what has worked and not worked within their unique perspectives as families who regularly encounter and interface with health and social systems. Parental experience should be central within discussions about the child’s plan of care, rather than silenced or suppressed (Currie & Szabo, 2019). Families can draw upon experiences and strengths, instead of focusing on rarity and what remains unknown.

Overall, these supportive efforts may reduce social exclusion and isolation for parents caring for children with rare NDDs.

## Conclusion

New interpretations of parenting and caregiving can mobilize appropriate health and social care supports and services for families caring for children with rare disorders. Validation of parent experiences reduces isolation and exclusion. Authentic regard for parent narratives honours parents’ experiences of meeting their children’s challenging care needs. Understanding parent experiences provides insight into systemic health and social support challenges faced by families and assist with the development of appropriate and supportive policies and services.

## Strengths and limitations

Hermeneutic phenomenology was utilized to explore stories of parents’ lived experiences of caring for children with rare NDDs. Although the sample size was small (which is typical for phenomenological studies), we were able to engage parents who had children with a range of rare NDDs. We also had fathers participating in the study which is often sparse in the literature. We did not validate the findings with the participants in the study but instead presented the research findings to stakeholders (including parents within local rare disease groups and HcPs), who acknowledged that the study outcomes aligned with their lived experiences. More narrative research is needed on parents’ perceptions of systemic, organizational power discourses, and humanizing policies in relation to caring for children with rare disorders.
